# permGPU: Using graphics processing units in RNA microarray association studies

**DOI:** 10.1186/1471-2105-11-329

**Published:** 2010-06-16

**Authors:** Ivo D Shterev, Sin-Ho Jung, Stephen L George, Kouros Owzar

**Affiliations:** 1 Department of Biostatistics and Bioinformatics, Duke University, 2424 Erwin Road, Durham, NC 27705, USA; 2 CALGB Statistical Center, Duke University, 2424 Erwin Road, Durham, NC 27705, USA

## Abstract

**Background:**

Many analyses of microarray association studies involve permutation, bootstrap resampling and cross-validation, that are ideally formulated as embarrassingly parallel computing problems. Given that these analyses are computationally intensive, scalable approaches that can take advantage of multi-core processor systems need to be developed.

**Results:**

We have developed a CUDA based implementation, permGPU, that employs graphics processing units in microarray association studies. We illustrate the performance and applicability of permGPU within the context of permutation resampling for a number of test statistics. An extensive simulation study demonstrates a dramatic increase in performance when using permGPU on an NVIDIA GTX 280 card compared to an optimized C/C++ solution running on a conventional Linux server.

**Conclusions:**

permGPU is available as an open-source stand-alone application and as an extension package for the R statistical environment. It provides a dramatic increase in performance for permutation resampling analysis in the context of microarray association studies. The current version offers six test statistics for carrying out permutation resampling analyses for binary, quantitative and censored time-to-event traits.

## Background

Many resampling algorithms used in microarray association studies can be formulated within the framework of embarrassingly parallel problems in the sense that the algorithm can be split up into smaller components which can be completed mutually independently of each other [1]. Standard algorithms used in this context include permutation and bootstrap resampling, and cross-validation. These algorithms consist of replicates which can be processed independently of each other. Furthermore, each replicate consists of the calculation of a large number of test statistics. The calculation of these test statistics can often be divided into independent parts. There are several protocols, including MPI [2] and OpenMP [3], that facilitate parallel programming for these algorithms.

Due to their highly parallel structure, Graphics Processing Units (GPU) are more effective than general-purpose Central Processing Units (CPU) for a set of algorithms widely used in the quantitative biomedical sciences. This has been demonstrated by using GPUs for example in feature detection in proteomics experiments [4], analysis of epistasis [5], statistical phylogenetics [6], and sequence alignment algorithms [7-10]. The R [11] extension package gputools [12] provides GPU enabled implementations of a set of commonly used functions for analysis of microarray data. Another attractive feature of using a GPU is that the hardware is relatively inexpensive, currently ranging from $400 to $800 for consumer grade cards (e.g., GTX 280, GTX 295) and $1200 to $2500 for high-end cards (e.g., Firestream 9270, Tesla C1060, Tesla C2050), compared to high-end multi-core workstations or cluster farms. GPU hardware can be easily added to existing workstations.

The primary focus of many published microarray association studies is the identification of genes differentially expressed with respect to a binary trait. An extensively cited example is the data set reported by Golub *et al *[13] who identified genes differentially expressed in Acute Myeloid Leukemia (AML) and Acute Lymphoblastic Leukemia (ALL). For many microarray experiments, especially those in cancer, the primary endpoint of interest is not a binary outcome but rather a censored time-to-event outcome such as time to death or time to relapse. An example is the data set reported by Beer *et al *[14] who identified genes associated with survival in early-stage Lung Adenocarcinoma. More recently, the Director's Challenge Consortium (DCC) for the Molecular Classification of Lung Adenocarcinoma reported predictive models for survival, based on gene expression profiles and clinical data from 442 patients with Adenocarcinoma Lung Cancer [15]. The Repository of Molecular Brain Neoplasia Data (Rembrandt) database currently provides 566 gene expression arrays and survival outcomes from brain cancer patients [16]. In this context, cases for which the event of interest is not realized (e.g., patients who are still alive) at the time of the analysis are censored. Specifically, let *Y*^0 ^denote the time of event and let *C *be the censoring time. What is observed is not *Y*^0 ^but rather *Y *= min{*Y*^0^, *C*} along with event indicator Δ = (*Y*^0 ^<*C*). The censoring times vary among patients since they are registered to studies at different times. A proper analysis must take into account not only the distribution of *Y*^0^, called the survival distribution, but also the censoring mechanism induced by the distribution of the censoring time *C*. A popular approach for analyzing time-to-event outcomes is to dichotomize the outcome at a given landmark, say *τ *> 0, that is believed to be clinically and biologically relevant [17]. For example, suppose that for a specific cohort of early stage Lung Adenocarcinoma patients, the median survival time *τ *= 5 years. In this case, one may categorize patients who live less than five years as high risk and those who live at least five years as low risk. This type of simplification allows for the use of methods and software tools developed for binary outcomes, but is not an optimal approach as the censoring mechanism is entirely ignored while the survival distribution is considered only at a single time-point (i.e., at *τ *= 5 years). In a recent article, Subramanian and Simon [17] conducted a critical review of sixteen published prognostic signatures in lung cancer and provided guidelines for statistical analysis in this context. Avoiding this type of binary transformation is the first item in this set of guidelines. Thus, software tools which can expeditiously conduct large-scale association testing for censored time-to-event outcomes are of great importance.

In this paper, we present a Compute Unified Device Architecture (CUDA) [18] framework, permGPU, that employs GPUs in microarray association studies. We illustrate the performance and applicability of permGPU within the context of permutation resampling for a number of test statistics. The software is provided as a stand-alone application that can be used to carry out permutation resampling inference for binary (e.g., case versus control), quantitative (e.g., blood pressure) or censored time-to-event (e.g., time to death) traits. For wider use, we also have integrated permGPU into the R statistical environment as an extension package. The source code along with documentation is provided in Additional File 1. Updates will be available from http://code.google.com/p/permgpu/.

## Implementation

We illustrate our framework using a simulation study by implementing a single-step multiple testing procedure based on the maximum statistic as described in [19] and [20]. The CUDA toolkit from NVIDIA, a minimal set of extensions to the C and C++ languages, is used for programming on a GTX 280 GPU, with 240 processor cores and 1 GB of memory. For comparison, we carry out a timing analysis based on a single CPU. The CPU code is compiled using g++ version 4.3.2 with -03 and -funroll-loops, while the GPU code is compiled using nvcc with -02 and --use_fast_math optimization flags. The current implementation is designed for CUDA enabled GPUs.

Both the GPU and CPU analyses are carried out on a 2.83 GHz Intel(R) Core(TM)2 Quad CPU Q9550 with 4 GB RAM of memory using the AMD64 Linux operating system. For wider applicability to the research community, we integrated permGPU into an R extension package. This implementation has been developed and tested on R version 2.10.1.

The gene-expression matrix ***X ***is of dimension *n *× *K*, where *K *is the number of genes, or other features, and *n *is the number of patients. The vector of outcomes is denoted by ***Y ***while the test statistic for testing the hypothesis of marginal association between feature *k *and the outcomes is denoted as *T*_*k*_. We consider test statistics where the critical region is of the form {|*T*_*k*_| > *ξ*} for some *ξ *> 0. For a given family-wise error rate (FWER) *α *∈ (0, 1), we determine the critical value *ξ *> 0 such that  under the hypothesis that no feature is associated with the outcome. The null sampling distribution is approximated using permutation resampling as follows:

1. Compute the *K *statistics *T*_1_, ..., *T*_*K *_based on ***Y***|***X***^1^, ..., ***Y ***|***X***^*K*^.

2. Let  be a random permutation of ***Y ***.

3. Compute , permutation replicates of the test statistics, based on |***X***^1^, ..., |***X***^*K*^.

4. Compute 

5. Repeat the last three steps *B - *1 additional times.

The unadjusted and FWER adjusted two-sided permutation *P*-values are computed as  and  respectively, where *I*[·] is the indicator function.

The code implementing our algorithm is a combination of kernels (GPU) and functions (CPU). While most of the calculations for the test statistics are carried out as kernels on the GPU, some of the calculations are relegated to functions on the CPU. The results of these functions are then copied to the GPU. For example, the random shuffling of the outcome is carried out only once per permutation. Therefore, we found it more efficient to permute the outcome vector on the CPU and then copy the result to the GPU. The components of the code that compute the *K *test statistics, their maximum and *P*-values, are separate kernels. The kernel that computes the *K *test statistics is the most computationally expensive. Global memory reads and computation are the primary bottlenecks for speed. To increase global memory read speed, we allocate ***X ***and other auxiliary data types via the function cudaMallocPitch(), thus automatically assuring aligned memory access. The random numbers are generated on the CPU. The data is copied between the CPU and GPU using standard CUDA library functions.

## Results

**Figure 1:**
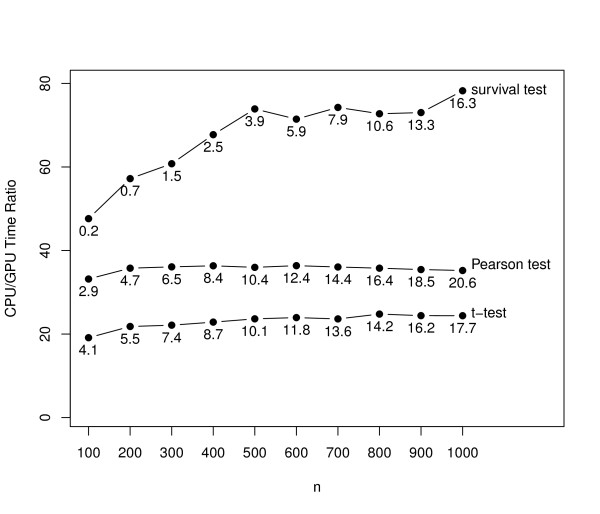
**CPU/GPU Time Ratios**. Illustration of the CPU/GPU time ratio as a function of *n*, for the *t*, Pearson and Cox rank score [21] tests for *K *= 60000. For the *t *and Pearson tests *B *= 10000 permutations are used while for the Cox rank score test *B *= 10 permutations are used. The GPU times (in seconds) are also shown. The results are based on one simulation replicate.

We illustrate the timing performance of our approach using an extensive simulation study considering the *t *test statistic, for two-sample problems, the Pearson test statistic, for continuous outcomes, and the Cox rank score test statistic [21], for censored time-to-event outcomes. The gene expression matrices are obtained by simulating *n *× *K *mutually independent and identically distributed standard normal variates where *n *= 100, ..., 1000 and *K *= 60000. For the two-sample case, the groups are drawn from a Bernoulli law with mean 0.5. For the continuous case, the outcomes are drawn from a standard normal law. For the time to event case, the expected censoring rate is set to 0.3. The illustrations for the *t *and Pearson test statistics are based on *B *= 10000 permutations. The CPU approach for the Cox rank score test statistic is prohibitively slow for large problems. For (*n, K*) = (1000, 60000), an analysis based on a mere *B *= 10 replicates takes approximately 21 minutes versus only 16 seconds on the GPU. The CPU/GPU execution time ratios along with the GPU times (measured in seconds) are shown in Figure 1. It can be seen that the biggest speed increase is in the case of the survival test, where speedup factors of 78 can be observed. Next, we illustrate an application of permGPU by conducting an analysis of the DCC [15] data set. For this illustration, we limit our attention to finding genes associated with survival. The analyses presented here are based on gene expression profiles and survival data from *n *= 442 patients from this data set. The observed death rate is 0.53 (236 out of 442) and the estimated median survival time is 70.5 months. The biospecimens are profiled on the Affymetrix GeneChip^® ^Human Genome U133A 2.0 array which profiles the RNA using *K *= 22283 probe sets. To conduct the analysis, we pre-processed the array source files (*.CEL) using the RMA algorithm [22]. We tested the association between the expression level of each of the *K *= 22283 probe sets with survival using the Cox score test with *B *= 10000 permutation replicates. In Table 1, we list the probe sets significant at most 0.05 two-sided FWER level. In addition to the observed test statistic, the unadjusted and FWER-adjusted permutation *P*-values, and the gene symbol and description linked to the probe set are provided. Using our GPU approach, the time required to conduct the analysis is about 20.1 minutes while the corresponding time based on the CPU approach is about 14.7 hours suggesting a time reduction factor in the order of 43.

**Table 1:** Association Analysis of DCC Data

probe set	T			Symbol	Description
220658_s_at	33.7	0e+00	0.004	ARNTL2	aryl hydrocarbon receptor nuclear translocator-like 2
221249_s_at	32.5	0e+00	0.006	FAM117A	family with sequence similarity 117, member A
218507_at	27.8	0e+00	0.018	C7orf68	chromosome 7 open reading frame 68
204524_at	27.2	0e+00	0.021	PDPK1	3-phosphoinositide dependent protein kinase-1
218498_s_at	27.2	0e+00	0.022	ERO1L	ERO1-like (S. cerevisiae)
208453_s_at	27.1	0e+00	0.022	XPNPEP1	X-prolyl aminopeptidase (aminopeptidase P) 1, soluble
201250_s_at	26.4	0e+00	0.026	SLC2A1	solute carrier family 2 (facilitated glucose transporter), member 1
200621_at	25.4	0e+00	0.036	CSRP1	cysteine and glycine-rich protein 1
210369_at	24.8	0e+00	0.043	SWAP70	SWAP switching B-cell complex 70 kDa subunit
205308_at	24.3	0e+00	0.049	FAM164A	family with sequence similarity 164, member A

Results from survival analysis of the Director's Challenge Consortium for the Molecular Classification of Lung Adenocarcinoma (DCC) data are shown in this table. The arrays were pre-processed using the RMA algorithm. The association between the summary expressions for each probe set and survival were tested using the Cox score test with *B *= 10000 permutation replicates. Probe sets significant at the two-sided FWER level of 0.05 are listed. The test statistic, permutation unadjusted and FWER adjusted *P*-values are denoted by *T*, , and  respectively. The gene symbol and description linked to each probe set was obtained from the Bioconductor hgu133a.db (version 2.3.5) annotation package.

## Discussion

Although we have limited the discussions to three tests, our approach can be applied to most tests used for analyzing high-dimensional data. Currently, our code also implements the Wilcoxon, Spearman and Cox score statistics, and can be extended using other test statistics including the family of score tests. The existing six test statistics can be used as a starting template. This may not be the most computationally efficient approach. It may however serve as an appropriate reference point. As in the case of the existing six test statistics, the most efficient code for any given test statistic must take full advantage of the computational resources offered by both the CPU and GPU and therefore should most likely consist of a combination of kernels and functions.

Permutation resampling to control FWER is one approach to address multiple testing for high-dimensional data. Our method can be easily extended to use the bootstrap via resampling with replacement. The false-discovery rate (FDR) [23] is another framework for adjusting for multiplicity. Our framework can be modified by omitting the calculation of the FWER adjusted *P*-values and applying any FDR algorithm to the unadjusted permutation *P*-values.

In many studies, including Shedden *et al *[15], the primary interest is not the identification of significant features but rather the building of predictive models. It is neither appropriate nor practical to build the model using all features. Feature selection is typically used to identify, from the training data, a set of features, which are marginally important based on some criterion. Note that the feature selection needs to be redone for each cross-validation training set. Our framework can be customized to speedup the feature selection by recomputing the test statistic *T*_*k *_based on the training set.

It may be argued that for microarray data sets the permutation analysis only needs to be done once and that the gain in speed is not practically important. In practice, however, one does not carry out a single analysis but rather a series of analyses to assess the implications of using a specific test statistic or pre-processing method. As a case in point, consider the analysis of the DCC data based on the Cox score statistic. As illustrated in [21], the test procedure is robust with respect to the survival distribution, but is not robust with respect to the marginal distributions of the gene expressions and is thus sensitive to outliers. The Cox rank score statistic [21], already implemented by permGPU, can be used to conduct a robust analysis. If considerable discrepancies between the sets of results from these two test statistics are observed, then one may need to be concerned about the presence of outliers in the microarray data. Furthermore, as pointed out in [24], microarray association analyses are sensitive to the pre-processing method used, especially in the presence of batch effects or outliers. For example, the DCC data set is comprised of specimens and clinical data provided by four institutions. Thus, one should be concerned not only about batch effects among the four sets of arrays but also about differences among the study populations and treatments. The conduct of, say, three additional analyses will bring the total time expenditure to almost 2.5 days in contrast to 1.5 hours on the GPU.

Finally, as illustrated in [25], for power and sample-size calculations, the permutation analysis needs to be repeated *N *times. Our approach can be extended to accommodate this type of analysis. For (*n, K*) = (600, 60000) and *B *= 10000, our GPU Pearson algorithm takes about 12 seconds. A power analysis based on *B *= 10000 and *N *= 1000 would then be expected to take less than 4 hours. Since the projected speedup time factor for this case is about 36, the expected time for completion on the CPU would exceed 5 days.

## Conclusions

A CUDA based implementation for deploying GPUs in RNA microarray association studies has been presented. Our implementation can be customized by incorporating other statistical tests and scales readily with GPU cores. An extension for incorporating our framework into the R statistical environment has been developed. Dramatic increase in speed in comparison to an optimized C/C++ code was demonstrated. The increased speed becomes more pronounced when the test statistic is computationally complex or the data set size is large, which makes our algorithm ideal for handling large genomic data sets. This is a practical framework that can be easily implemented using relatively inexpensive hardware.

## Availability and requirements

• **Project name**: permGPU

• **Project home page**: http://code.google.com/p/permgpu/

• **Operating system**: Linux AMD64

• **Programming language**: CUDA, C/C++ and R

• **Other requirements**: CUDA SDK and Toolkit 2.0 or higher; gcc/g++ 4.3.2; R http://www.R-project.org 2.10.1; Biobase http://www.bioconductor.org 2.6.1

• **License**: GPL v3

## Abbreviations

CPU: Central Processing Unit; CUDA: Compute Unified Device Architecture; GPL: General Public License; GPU: Graphics Processing Unit; FDR: False Discovery Rate; FWER: Family-Wise Error Rate; GTX 280: NVIDIA GeForce GTX 280; MPI: Message Passing Interface; OpenMP: Open Multi-Processing; RNA: Ribonucleic Acid; SDK: Software Development Kit.

## Competing interests

The authors declare that they have no competing interests.

## Authors' contributions

IDS conceptualized the research, designed, programmed, optimized and tested the algorithm, and drafted the manuscript; S-HJ contributed to the research and critically revised the manuscript; SLG contributed to the research, provided funding and critically revised the manuscript; KO proposed and conceptualized the research, and drafted the manuscript. All authors read and approved the final manuscript.

## Supplementary Material

Additional file 1 **Supplementary Material for: "**permGPU**: Using graphics processing units in RNA microarray association studies"**. The compressed tar archive contains the source code for the examples discussed in "permGPU: Using graphics processing units in RNA microarray association studies" by Shterev et al. It also contains a tutorial for compiling and executing the code. The development version of the code is available for download from http://code.google.com/p/permgpu/. Click here for file

## References

[B1] Foster I Designing and Building Parallel Programs: Concepts and Tools for Parallel Software Engineering 1995 Addison-Wesley

[B2] The Message Passing Interface (MPI) standard http://www.mcs.anl.gov/research/projects/mpi/

[B3] The OpenMP API specification for parallel programming http://openmp.org/wp/

[B4] Hussong R Gregorius B Tholey A Hildebrandt A Highly accelerated feature detection in proteomics data sets using modern graphics processing units Bioinformatics 2009 25 1937 1943 10.1093/bioinformatics/btp294 19447788

[B5] Sinnott-Armstrong NA Greene CS Cancare F Moore JH Accelerating epistasis analysis in human genetics with consumer graphics hardware BMC Bioinformatics 2009 2 10.1186/1756-0500-2-149 PMC2732631 19630950

[B6] Suchard MA Rambaut A Many-core algorithms for statistical phylogenetics Bioinformatics 2009 25 1370 1376 10.1093/bioinformatics/btp244 19369496 PMC2682525

[B7] Schatz MC Trapnell C Delcher AL Varshney A High-throughput sequence alignment using Graphics Processing Units BMC Bioinformatics 2007 8 10.1186/1471-2105-8-474 18070356 PMC2222658

[B8] Manavski S Valle G CUDA compatible GPU cards as efficient hardware accelerators for Smith-Waterman sequence alignment BMC Bioinformatics 2008 9 10.1186/1471-2105-9-S2-S10 18387198 PMC2323659

[B9] Jung S Parallelized pairwise sequence alignment using CUDA on multiple GPUs BMC Bioinformatics 2009 10 10.1186/1471-2105-10-164

[B10] Liu Y Maskell DL Schmidt B CUDASW++: Optimizing Smith-Waterman sequence database searches for CUDA-enabled graphics processing units BMC Bioinformatics 2009 2 10.1186/1756-0500-2-73 PMC2694204 19416548

[B11] R Development Core Team R: A Language and Environment for Statistical Computing 2009 R Foundation for Statistical Computing, Vienna, Austria http://www.R-project.org [ISBN 3-900051-07-0]

[B12] Buckner J Wilson J Seligman M Athey B Watson S Meng F The gputools package enables GPU computing in R Bioinformatics 2010 26 134 135 10.1093/bioinformatics/btp608 19850754 PMC2796814

[B13] Golub T Slonim D Tamayo P Huard C Gaasenbeek M Mesirov J Coller H Loh M Downing J Caligiuri M Bloomfield C Lander E Molecular Classification of Cancer: Class discovery and class prediction by gene expression monitoring Science 1999 286 5439 531 537 10.1126/science.286.5439.531 10521349

[B14] Beer DG Kardia SLR Huang CC Giordano TJ Levin AM Misek DE Lin L Chen G Gharib TG Thomas DG Lizyness ML Kuick R Hayasaka S Taylor JMG Iannettoni MD Orringer MB Hanash S Gene-expression profiles predict survival of patients with lung adenocarcinoma Nat Med 2002 8 8 816 824 12118244 10.1038/nm733

[B15] for the Molecular Classification of Lung Adenocarcinoma DCC Shedden K Taylor JMG Enkemann SA Tsao MS Yeatman TJ Gerald WL Eschrich S Jurisica I Giordano TJ Misek DE Chang AC Zhu CQ Strumpf D Hanash S Shepherd FA Ding K Seymour L Naoki K Pennell N Weir B Verhaak R Ladd-Acosta C Golub T Gruidl M Sharma A Szoke J Zakowski M Rusch V Kris M Viale A Motoi N Travis W Conley B Seshan VE Meyerson M Kuick R Dobbin KK Lively T Jacobson JW Beer DG Gene expression-based survival prediction in lung adenocarcinoma: a multi-site, blinded validation study Nat Med 2008 14 8 822 827 10.1038/nm.1790 18641660 PMC2667337

[B16] Madhavan S Zenklusen J Kotliarov Y Sahni H Fine H Buetow K Rembrandt: helping personalized medicine become a reality through integrative translational research Mol Cancer Res 2009 7 2 157 167 10.1158/1541-7786.MCR-08-0435 19208739 PMC2645472

[B17] Subramanian J Simon R Gene expression-based prognostic signatures in lung cancer ready for clinical use? J Natl Cancer Inst 2010 102 1 11 10.1093/jnci/djp493 PMC2902824 20233996

[B18] NVIDIA Compute unified device architecture (CUDA) programing guide 2008 [Version 2.2]

[B19] Westfall PH Young SS Resampling-Based Multiple Testing: Examples and Methods for P-value Adjustment 1993 New York: Wiley-Interscience

[B20] Ge Y Dudoit S Speed TP Resampling-based multiple testing for microarray data analysis TEST 2003 12 1 44 10.1007/BF02595811

[B21] Jung SH Owzar K George SL A multiple testing procedure to associate gene expression levels with survival Statistics in Medicine 2005 24 3077 3088 10.1002/sim.2179 16189805

[B22] Irizarry R Hobbs B Collin F Beazer-Barclay Y Antonellis K Scherf U Speed T Exploration, normalization, and summaries of high density oligonucleotide array probe level data Biostatistics 2003 4 2 249 264 10.1093/biostatistics/4.2.249 12925520

[B23] Benjamini Y Hochberg Y Controlling the false discovery rate: A practical and powerful approach to multiple testing JR Statist Soc B 1995 57 289 300

[B24] Owzar K Barry WT Jung SH Sohn I George SL Statistical challenges in preprocessing in microarray experiments in cancer Clin Cancer Res 2008 14 19 5959 5966 10.1158/1078-0432.CCR-07-4532 18829474 PMC3529914

[B25] Jung SH Bang H Young SS Sample size calculation for multiple testing in microarray data analysis Biostatistics 2005 6 157 169 10.1093/biostatistics/kxh026 15618534

