# Supplementary material for: permGPU: Using graphics processing units in RNA microarray association studies
Source: BMC Bioinformatics. 2010 Jun 16;11:329. doi: 10.1186/1471-2105-11-329 (PMC2910023; doi:10.1186/1471-2105-11-329)
Supplement: Additional file 1 — Supplementary Material for: "permGPU: Using graphics processing units in RNA microarray association studies". The compressed tar archive contains the source code for the examples discussed in "permGPU: Using graphics processing units in RNA microarray association studies" by Shterev et al. It also contains a tutorial for compiling and executing the code. The development version of the code is available for download from http://code.google.com/p/permgpu/. [file 1471-2105-11-329-S1.GZ › permGPU-supp-material/dchallenge-preprocessing.pdf]

Supplementary Information for "permGPU: Using graphics processing units in RNA microarray association studies"

Ivo D. Shterev   Sin-Ho Jung   Stephen L. George   Kouros Owzar

June 2, 2010

1 Introduction

This supplemental document provides instructions for replicating the pre-processing steps analyzing the Director's Challenge Consortium lung cancer data [Shedden *et al*, 2008] in "permGPU: Using graphics processing units in RNA microarray association studies" by [Shterev *et al*, 2010]. The final output files are available for download from `http://code.google.com/p/permgpu/`.

2 Data Import

The phenotypic and genotypic data were obtained from `https://array.nci.nih.gov/caarray/project/jacob-00182`. This data set consists of 443 CEL files. The phenotypic data are provided by a file called `experiment-id-1015945236141280.all.sdrf` For convenience, we rename the observed survival time and event indicator variables as `ostime` and `event` respectively. The latter is recoded as 1 ("Dead") or 0 ("Alive"). The row names of this data object are set to CEL file names.

```
> ROOTDIR = "/srv/DCC/"
> foodir = function(dir, rdir = ROOTDIR) {
+     paste(rdir, dir, sep = "")
+ }
> CELDIR = foodir("CEL")
> PROCDIR = foodir("PROC")
> phfile = foodir("experiment-id-1015945236141280.all.sdrf")
> pdat = read.table(phfile, sep = "\t", header = TRUE, na.strings = c("na",
+     "", " "))
> tools::md5sum(phfile)
```

```
/srv/DCC/experiment-id-1015945236141280.all.sdrf
              "a78f9c4196b296e45507f2b7da9672b1"
```

```
> pdat <- data.frame(pdat, ostime = pdat[["Characteristics.MONTHS_TO_LAST_CONTACT_OR_DEATH."]],
+     event = ifelse(pdat[["Characteristics.VitalStatus."]] ==
+         "Dead", 1, 0))
> rownames(pdat) = as.character(pdat[["Array.Data.File"]])
```

3 Pre-processing

Next, we import the CEL files into R. As a first step, we check to make sure that the set of CEL file names according to `pdat` coincides with the set of physical CEL file names.

```
> library(affy)
> library(hgu133a.db)
> if (setequal(rownames(pdat), list.celfiles(CELDIR))) {
+     CELDAT = ReadAffy(filenames = rownames(pdat), celfile.path = CELDIR)
+     pData(CELDAT) = pdat
+ }
```

One of the patients has missing survival data. This patient is excluded from all analyses as follows:

```
> CELDAT = CELDAT[, !is.na(pdat$ostime)]
```

Next, we pre-process the raw data using the RMA [Irizarry *et al*, 2003] algorithm as follows:

```
> RMADAT = rma(CELDAT)

Background correcting
Normalizing
Calculating Expression
```

Next, the pre-processed data are saved as an `R` object to a physical file. This file can be used to carry out association analyses using the `permGPU` `R` extension package.

```
> save(RMADAT, file = foodir("RMADAT-DCHALL.Rdata"))
> tools::md5sum(foodir("RMADAT-DCHALL.Rdata"))

      /srv/DCC/RMADAT-DCHALL.Rdata
"8dd297ab6a56946d6842b04b21df37b4"
```

Finally, we write the `R` data objects to physical files suitable for the standalone GPU code use:

```
> write.csv(exprs(RMADAT), paste(ROOTDIR, "RMADAT-EXP.csv", sep = ""),
+     quote = FALSE, row.names = FALSE)
> write.csv(data.frame(patid = rownames(pData(RMADAT)), otime = pData(RMADAT)$ostime,
+     event = pData(RMADAT)$event), paste(ROOTDIR, "RMADAT-PHENO.csv",
+     sep = ""), quote = FALSE, row.names = FALSE)
> tools::md5sum(paste(ROOTDIR, "RMADAT-EXP.csv", sep = ""))

           /srv/DCC/RMADAT-EXP.csv
"ffeba8e06ba30258cfb0b90b5816b617"

> tools::md5sum(paste(ROOTDIR, "RMADAT-PHENO.csv", sep = ""))

         /srv/DCC/RMADAT-PHENO.csv
"6966c490d46624b2c63660f76e712cde"
```

4 Software Version

Package version information is provided next.

```
> sessionInfo()

R version 2.10.1 (2009-12-14)
x86_64-pc-linux-gnu

locale:
 [1] LC_CTYPE=en_US.UTF-8       LC_NUMERIC=C
 [3] LC_TIME=en_US.UTF-8        LC_COLLATE=en_US.UTF-8
```

```
 [5] LC_MONETARY=C              LC_MESSAGES=en_US.UTF-8
 [7] LC_PAPER=en_US.UTF-8       LC_NAME=C
 [9] LC_ADDRESS=C               LC_TELEPHONE=C
[11] LC_MEASUREMENT=en_US.UTF-8 LC_IDENTIFICATION=C

attached base packages:
[1] stats     graphics  grDevices utils     datasets  methods   base

other attached packages:
[1] hgu133acdf_2.4.0    hgu133a.db_2.3.5    org.Hs.eg.db_2.3.6
[4] RSQLite_0.8-4       DBI_0.2-5           AnnotationDbi_1.8.2
[7] affy_1.24.2         Biobase_2.6.1

loaded via a namespace (and not attached):
[1] affyio_1.14.0        preprocessCore_1.6.0 tools_2.10.1
```

References

[Jung *et al*, 2005] Jung, S.-H., Owzar K., George, S.L. (2005) A multiple testing procedure to associate gene expression levels with survival. *Statistics in Medicine.* **24(20)**, 3077–88.

[Shterev *et al*, 2010] Shterev, I.D., Jung, S.-H., George, S.L., Owzar, K. (2010) `permGPU`: Using graphics processing units in RNA microarray association studies. Duke Biostatistics Working Paper Series.

[Shedden *et al*, 2008] For the Director's Challenge Consortium for the Molecular Classification of Lung Adenocarcinoma, Shedden K, Taylor JMG, Enkemann SA, Tsao MS, Yeatman TJ, Gerald WL, Eschrich S, Jurisica I, Giordano TJ, Misek DE, Chang AC, Zhu CQ, Strumpf D, Hanash S, Shepherd FA, Ding K, Seymour L, Naoki K, Pennell N, Weir B, Verhaak R, Ladd-Acosta C, Golub T, Gruidl M, Sharma A, Szoke J, Zakowski M, Rusch V, Kris M, Viale A, Motoi N, Travis W, Conley B, Seshan VE, Meyerson M, Kuick R, Dobbin KK, Lively T, Jacobson JW, Beer DG (2008) Gene expression-based survival prediction in lung adenocarcinoma: a multi-site, blinded validation study. *Nat Med.* **14**(8):822–827.

[Irizarry *et al*, 2003] Irizarry RA, Hobbs B, Collin F, Beazer-Barclay YD, Antonellis KJ, Scherf U, Speed TP (2003) Exploration, normalization, and summaries of high density oligonucleotide array probe level data. *Biostatistics.* **4**(2):249-264.
