# Supplementary material for: permGPU: Using graphics processing units in RNA microarray association studies
Source: BMC Bioinformatics. 2010 Jun 16;11:329. doi: 10.1186/1471-2105-11-329 (PMC2910023; doi:10.1186/1471-2105-11-329)
Supplement: Additional file 1 — Supplementary Material for: "permGPU: Using graphics processing units in RNA microarray association studies". The compressed tar archive contains the source code for the examples discussed in "permGPU: Using graphics processing units in RNA microarray association studies" by Shterev et al. It also contains a tutorial for compiling and executing the code. The development version of the code is available for download from http://code.google.com/p/permgpu/. [file 1471-2105-11-329-S1.GZ › permGPU-supp-material/tutorial.pdf]

Supplementary Information for "permGPU: Using graphics processing units in RNA microarray association studies"

Ivo D. Shterev Sin-Ho Jung Stephen L. George Kouros Owzar

March 22, 2010

1 Introduction

This supplemental document provides instructions for compiling and executing the code for the examples discussed in "permGPU: Using graphics processing units in RNA microarray association studies" by [Shterev *et al*, 2010]. The source code for building both a stand-alone application and an R package for the R statistical environment are available under a GPL-v3 license from `http://code.google.com/p/permgpu/`. The R package has its own internal documentation. It can be installed at the UNIX prompt as follows

```
$ R CMD INSTALL permGPU_0.11.tar.gz
```

The instructions provided in this document are primarily intended for building the stand-alone application.

2 Listing and Description of Files

2.1 Main Files

The CUDA source code for conducting analyses of study data (using t, Wilcoxon, Pearson, Spearman, Cox score or Cox Rank Score [Jung *et al*, 2005] tests) is contained in the file `perm_test_all_s.cu` which is provided as part of the supplemental material. The t test is computed assuming unequal variances in the two groups. Additionally, the CUDA source code for conducting timing analyses for the t, Pearson and Cox rank score tests are provided. These are:

- `perm_t_test_time.cu` (for the t test)
- `perm_pearson_test_time.cu` (for the Pearson test)
- `perm_survival_test_time.cu` (for the survival test)

For each of these three cases, the $K \times n$[1] expression matrix is simulated based on nK independently and identically distributed standard normal variates. For the t test, the grouping vector Y is simulated from a Bernoulli law with mean 0.5. As such, the two groups are on average of the same size. For the Pearson test, the response vector is simulated from a standard normal law. For the survival test, the observed times are simulated from a standard half-normal law while the event indicators are simulated from a Bernoulli law with mean 0.7. As such, the expected censoring rate is 0.3. These can be easily modified for other scenarios. For example, in the case of right-censored clinical

[1] The maximum value of n for the current version of GPU codes is 1000.

time to event data (e.g., overall survival), the actual time to event distribution can be drawn from an exponential law while the censoring times can be drawn from a uniform law. The corresponding observed times and event indicators can then be obtained from these.

Each timing analysis is based on n patients, K markers and B permutation replicates, each to be specified by the user. The execution time is echoed to the terminal after completion of the analysis. The execution time takes into account the time expended on

- Copying the data from the CPU to the GPU (the data are simulated on the CPU)
- Calculation of the observed test statistics
- Calculation of the unadjusted and FWER adjusted P-values
- Copying the results (test statistics and P-values) from the GPU to the CPU

2.2 Support Files

The following files, taken from `http://www.agner.org/random/`, are used in the random number generation on the CPU.

1. `randomc.h`
2. `stocc.h`
3. `mersenne.cpp`
4. `stoc1.cpp`
5. `userintf.cpp`

`http://sites.google.com/site/jivsoft/Home/compute-ranks-of-elements-in-a-c---array-or-vector`. This is the web page for the following template file used in computing the ranks of a vector of elements on the CPU.

1. `ranker.h`

3 Compilation and Execution

3.1 Timing Analysis

The CUDA source code for the t-test timing analyses (`perm_t_test_time.cu`) is compiled as follows:

```
nvcc -O2 --use_fast_math perm_t_test_time.cu -o permGPU
```

First, the paths to the CUDA binaries and libraries need to be configured appropriately. Assuming that these are installed under `/usr/local`, this can be accomplished (on a BASH shell) as follows:

```
export PATH=$PATH:/usr/local/cuda/bin:/usr/local/cuda/cudaprof/bin/
export LD_LIBRARY_PATH=$LD_LIBRARY_PATH:/usr/local/cuda/lib
```

The corresponding CPU source code is compiled as follows:

```
g++ -funroll-loops -O3 perm_t_test_timeCPU.cpp -o permCPU
```

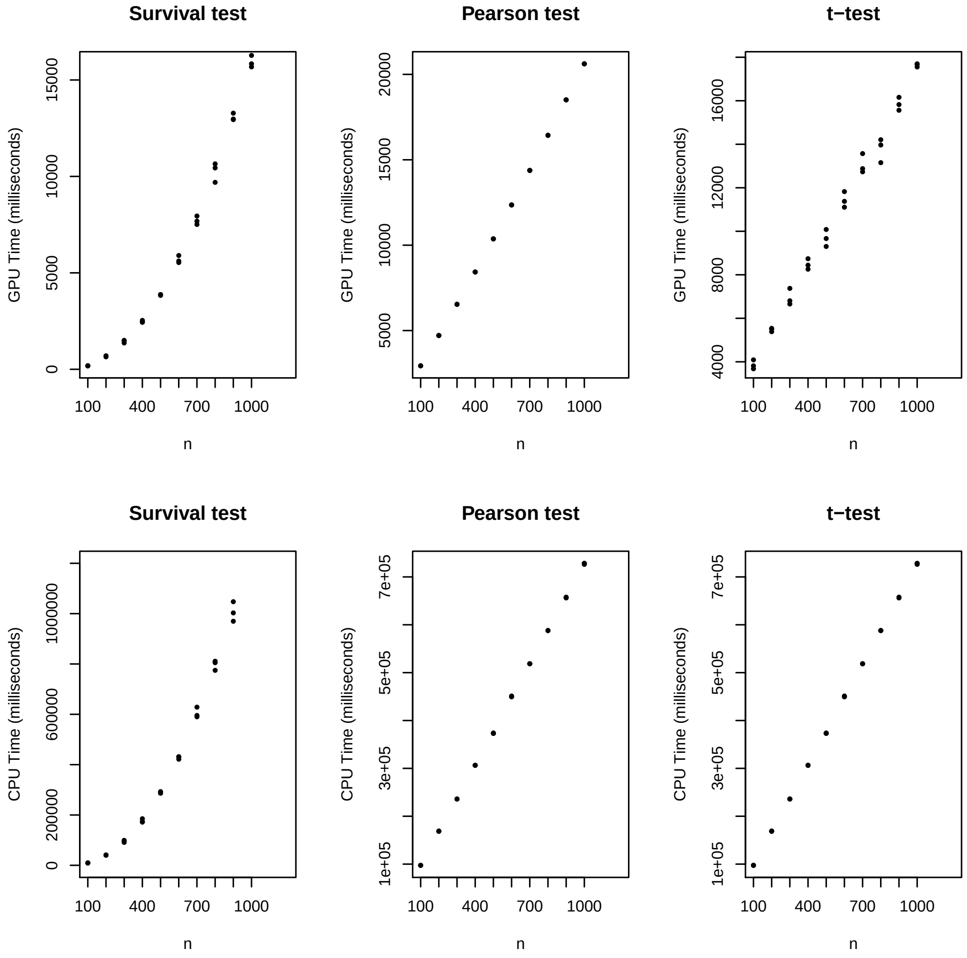

Figure 1: Illustration of GPU and CPU timing for the t, Pearson and survival tests. For the t and Pearson tests $B = 10000$ permutation replicates are generated. For the survival test only $B = 10$ permutation replicates are generated.

To conduct the timing analysis based on say $n = 700$ patients, $K = 60,000$ markers and $B = 10,000$ permutations, based on the binary generated from the CUDA or CPU source code, run respectively:

```
./permCPU 700 60000 10000
./permGPU 700 60000 10000
```

Additional timing results for the t-test, Pearson test and survival test, for both the GPU and CPU are shown in Fig. 1. For each (n, K) pair three independent execution replicates are generated. For the t and Pearson tests $B = 10000$ permutation replicates were generated. For the survival test only $B = 10$ permutation replicates were generated.

3.2 Data Analysis

To conduct an analysis of a data set, the code in `perm_test_all_s.cu` is to be used. Analogous to the CUDA timing code, it is compiled as follows:

```
nvcc -O2 --use_fast_math perm_test_all_s.cu -o permGPU
```

To execute the generated binary, the user has to provide five arguments:

- `expfile`: This argument refers to a physical file containing the expression data. This file consists of $K + 1$ rows and $n + 1$ columns. The first column contains the marker ids (e.g, probe set ids in the case of Affymetrix RNA expression arrays) while the first row contains the patient or sample ids. This should be a comma delimited file.

- `phenofile`: This argument refers to a physical file containing the outcome data. The first column contains the sample (patient) id labels. For the t and Wilcoxon tests, the second column contains 0 or 1s. For the Pearson and Spearman tests, the second column consists of numeric entries. For the survival test, the second column consists of positive numeric entries while the third column consists of 0 or 1s. These should be comma delimited files. The first row of each file is used as the header.

- `test`: This argument specifies the test to be used. The possible choices are `ttest, wilcoxon, pearson, spearman, cox` or `npcox`.

- `outfile`: This refers to a physical file containing the output. It consists of a header, K rows (one row per marker) and four columns: The marker ids, the unadjusted permutation P-value, the FWER adjusted permutation P-value and the test statistic.

- `B`: Number of permutations.

The code is executed as follows:

```
./permGPU expfile phenofile test outfile B
```

3.2.1 Cox or Cox Rank Score Tests

Let us consider a survival analysis example. We will use the `R` statistical environment to generate the files `expfile` and `phenofile`. We begin by generating event and censoring times from an exponential law, with parameter log(2), and uniform law on the interval $[1, 2]$. We also generate a gene expression matrix of dimension $K \times n$ from identically and mutually independent normal variates.

```
set.seed(123)
n=200
K=60000
atime=rexp(n,log(2))
ctime=runif(n,1,2)
otime=pmin(atime,ctime)
event=as.integer(atime<ctime)
PHENO=data.frame(otime,event)
EXP=matrix(rnorm(n*K),K,n)
```

Next, we generate dummy patient and gene ids and assign them to the phenotype and expression data objects:

```
patid=paste("pt",1:n,sep="")
gid=paste("gid",1:K,sep="")
rownames(PHENO)=patid
rownames(EXP)=gid
colnames(EXP)=patid
```

Next, we write these objects to physical files:

```
write.csv(EXP,"NPCOX-EXP-example.csv",quote=FALSE)
write.csv(PHENO,"NPCOX-PHENO-example.csv",quote=FALSE)
```

To carry out the analysis using the `cox` test with $B = 10000$:

```
./permGPU NPCOX-EXP-example.csv NPCOX-PHENO-example.csv cox p_P_T.txt 10000
```

To carry out the analysis using the `npcox` test with $B = 10000$:

```
./permGPU NPCOX-EXP-example.csv NPCOX-PHENO-example.csv npcox p_P_T.txt 10000
```

The results are put into a file called `p_P_T.txt`.

3.2.2 t or Wilcoxon Tests

To illustrate an analysis based on the t or Wilcoxon tests, we will use the Golub data provided by the Bioconductor package `golubEsets`.

```
library(golubEsets)
library(Biobase)
data(Golub_Merge)
PHENO=data.frame(y=ifelse(pData(Golub_Merge)[["ALL.AML"]]=="ALL",0,1))
EXP=exprs(Golub_Merge)
```

We proceed by writing the objects to physical files:

```
write.csv(EXP,"BINARY-EXP-example.csv",quote=FALSE)
write.csv(PHENO,"BINARY-PHENO-example.csv",quote=FALSE)
```

To carry out the analysis using the t-test with $B = 10000$:

```
./permGPU BINARY-EXP-example.csv BINARY-PHENO-example.csv ttest p_P_T.txt 10000
```

To carry out the analysis using the Wilcoxon test with $B = 10000$:

```
./permGPU BINARY-EXP-example.csv BINARY-PHENO-example.csv wilcoxon p_P_T.txt 10000
```

3.2.3 Pearson or Spearman Tests

We illustrate the analysis based on the Pearson or Spearman tests by generating the gene expression matrix and the outcomes from independent and identically distributed standard normal variates.

```
set.seed(123)
n=200
K=60000
PHENO=data.frame(y=rnorm(n))
EXP=matrix(rnorm(n*K),K,n)
```

Next, we generate dummy patient and gene ids and assign them to the phenotype and expression data objects:

```
patid=paste("pt",1:n,sep="")
gid=paste("gid",1:K,sep="")
rownames(PHENO)=patid
rownames(EXP)=gid
colnames(EXP)=patid
```

We proceed by writing the objects to physical files:

```
write.csv(EXP,"NUMERIC-EXP-example.csv",quote=FALSE)
write.csv(PHENO,"NUMERIC-PHENO-example.csv",quote=FALSE)
```

To carry out the analysis using the Pearson test with $B = 10000$:

```
./permGPU NUMERIC-EXP-example.csv NUMERIC-PHENO-example.csv pearson p_P_T.txt 10000
```

To carry out the analysis using the Spearman test with $B = 10000$:

```
./permGPU NUMERIC-EXP-example.csv NUMERIC-PHENO-example.csv spearman p_P_T.txt 10000
```

4 Application of `permGPU` to a Real Data Set

Next, we provide a description of applying `permGPU` to the Director's Challenge Consortium (DCC) for the Molecular Classification of Lung Adenocarcinoma data set [Shedden *et al*, 2008]. The preprocessing steps are outlined in a separate document `dchallenge-preprocessing.pdf`. The resulting files are `RMADAT-EXP-example.csv` (summary expression based on the RMA [Irizarry *et al*, 2003] algorithm) and `RMADAT-PHENO-example.csv` (the phenotype data). These files can be replicated using the instructions provided in the supplemntary documentation file (`dchallenge-preprocessing.pdf`) or downloaded from the project webpage. The Cox score and Cox rank score tests using $B = 10000$ permutations can be carried out respectively:

```
./permGPU RMADAT-EXP-example.csv RMADAT-PHENO-example.csv cox DCCcox.txt 10000
./permGPU RMADAT-EXP-example.csv RMADAT-PHENO-example.csv npcox DCCnpcox.txt 10000
```

References

[Jung *et al*, 2005] Jung SH, Owzar K, George SL (2005) A multiple testing procedure to associate gene expression levels with survival. *Statistics in Medicine.* **24(20)**, 3077–88.

[Shterev *et al*, 2010] Shterev ID, Jung SH, George SL, Owzar K (2010) `permGPU`: Using graphics processing units in RNA microarray association studies. Duke Biostatistics Working Paper Series.

[Shedden *et al*, 2008] For the Director's Challenge Consortium for the Molecular Classification of Lung Adenocarcinoma, Shedden K, Taylor JMG, Enkemann SA, Tsao MS, Yeatman TJ, Gerald WL, Eschrich S, Jurisica I, Giordano TJ, Misek DE, Chang AC, Zhu CQ, Strumpf D, Hanash S, Shepherd FA, Ding K, Seymour L, Naoki K, Pennell N, Weir B, Verhaak R, Ladd-Acosta C, Golub T, Gruidl M, Sharma A, Szoke J, Zakowski M, Rusch V, Kris M, Viale A, Motoi N, Travis W, Conley B, Seshan VE, Meyerson M, Kuick R, Dobbin KK, Lively T, Jacobson JW, Beer DG (2008) Gene expression-based survival prediction in lung adenocarcinoma: a multi-site, blinded validation study. *Nat Med.* **14**(8):822–827.

[Irizarry *et al*, 2003] Irizarry RA, Hobbs B, Collin F, Beazer-Barclay YD, Antonellis KJ, Scherf U, Speed TP (2003) Exploration, normalization, and summaries of high density oligonucleotide array probe level data. *Biostatistics.* **4**(2):249-264.
